# Beyond Precision: Why the Future of Robotic Joint Arthroplasty Demands Continued Research

**DOI:** 10.1016/j.artd.2026.102071

**Published:** 2026-06-13

**Authors:** Matthew Bullock, Jonathan R. Danoff

**Affiliations:** a Department of Orthopaedic Surgery, Joan C. Edwards School of Medicine, Marshall University, Huntington, WV, USA; b Department of Orthopaedic Surgery, North Shore University Hospital/Long Island Jewish Medical Center, Northwell Health, Manhasset, NY, USA

**Keywords:** Robotic revision, Hip arthroplasty, Knee arthroplasty, Efficiency, Joint line

## Introduction

As the demand for joint replacement surgery continues to rise, driven by expanding indications and an increasingly active, aging population, surgeons and health systems are expected to deliver consistent, high-quality outcomes across diverse practice environments [1,2]. Meeting this challenge requires surgical technologies that are efficient, reproducible, adaptable, and durable while supporting implant survivorship. Although robotic assistance has emerged as a promising tool to advance these goals, its clinical role remains incompletely defined. While smaller cohort studies suggest improvements in safety and efficiency, high-quality registry analyses have consistently demonstrated no superiority in clinical or patient-reported outcomes compared with conventional techniques [3,4]. Similarly, large registry studies have shown no reduction in revision risk or improvement in midterm survivorship [4,5]. Accordingly, continued rigorous investigation is needed to clarify the clinical scenarios in which robotic assistance adds value, to refine the technology, and to determine how potential benefits can be extended to a broader patient population.

This viewpoint examines robotic-assisted arthroplasty from the perspective of surgeons who have transitioned from conventional manual techniques during ongoing changes in training practices and technological adoption. These shifts include 3-dimensional strategies for joint line reconstruction, dynamic ligamentous and gap balancing with real-time visual feedback, and the expanding application of robotic assistance in revision surgery. Although early evidence suggests gains in precision and reproducibility, further investigation is necessary to determine how these technical advantages translate into long-term clinical outcomes, cost-effectiveness, operative efficiency, and broader system-level impact.

## Expanding access and reducing variability

According to the 2025 American Joint Replacement Registry report [6], utilization of robotic assistance continues to increase in knee arthroplasty. McAuliffe et al. were among the first to demonstrate lower revision rates among low-volume surgeons using robotic assistance, with outcomes comparable to those of high-volume surgeons employing conventional instrumentation [7]. These findings suggest that robotic assistance may partially mitigate volume-related disparities among surgeons performing fewer than 50 TKAs annually.

Similarly, Lee et al. [8] reported that low-volume surgeons using robotic assistance achieved perioperative and longer-term outcomes comparable to those of high-volume surgeons utilizing conventional techniques, with perioperative outcomes also similar to those observed among nonrobotic high-volume surgeons. Collectively, these studies suggest robotic assistance may attenuate outcome differences associated with case volume; however, the durability and generalizability of these findings require further investigation.

Robotic platforms may reduce surgical variability by providing intraoperative guidance, standardized workflows, and objective data. Prior studies have demonstrated improvements in implant alignment, resection accuracy, and soft tissue balance with robotic systems compared with conventional jig-based techniques [2,9,10]. These findings do not diminish the ability of experienced surgeons using conventional techniques to achieve excellent outcomes; rather, robotic assistance may help narrow performance gaps by promoting greater reproducibility and precision [8,10,11]. Whether these technical advantages ultimately translate into lower revision rates, improved functional outcomes, or greater patient satisfaction remains an important and unresolved question. Importantly, robotic assistance should be considered an adjunct to, rather than a replacement for, surgical experience, as optimal outcomes continue to depend on intraoperative judgment, tactile assessment, and the infrastructure of high-volume surgeons and centers.

## Objectivity, precision, and the limits of “feel”

Traditional arthroplasty techniques rely heavily on surgeon experience and tactile feedback, introducing an element of subjectivity into intraoperative decision-making [12]. In contrast, robotic systems reduce reliance on estimation by incorporating digital measurement, translating intraoperative “feel” into quantifiable data [10]. In knee arthroplasty, modern platforms enable surgeons to plan implant positioning relative to native anatomy and mechanical axes while accommodating a range of alignment philosophies, including mechanical, kinematic, and hybrid approaches.

Robotic platforms have demonstrated accuracy within 1 mm and 1 degree, highlighting the level of precision achievable with these technologies [13]. This degree of consistency may be particularly valuable in complex primary cases and revision cases, where anatomic landmarks are distorted and margins for error are limited. Nonetheless, further investigation is required to determine the extent to which increased precision is clinically meaningful and how incremental gains influence long-term implant survivorship.

## Implications for training and workforce development

Orthopaedic training is undergoing a generational shift, similar to transitions previously observed with adoption of arthroscopic techniques. Although recent graduates report increasing exposure to robotic platforms, opportunities to develop proficiency with conventional techniques may be declining, and the long-term implications of this shift remain unclear [12,14]. As adoption expands, robotic systems may increasingly be regarded as standard surgical tools rather than adjuncts to conventional techniques.

In a national survey of graduating residents by Duensing et al., 70% of trainees reported exposure to robotic-assisted total knee arthroplasty (TKA) [14]. A substantial proportion of respondents perceived improved procedural understanding with robotic assistance; however, perceptions regarding improvements in clinical outcomes were mixed. Notably, 25% indicated that robotic use negatively affected their training with conventional instrumentation, and greater robotic exposure was associated with reduced comfort performing traditional TKA. Collectively, these findings suggest that while robotic assistance may support certain aspects of surgical education, balanced training that ensures continued proficiency with manual instrumentation remains essential.

From an educational perspective, robotic assistance may facilitate instruction in complex concepts such as alignment strategies, soft tissue balancing, and component positioning through enhanced visualization and real-time intraoperative feedback [12,14]. However, whether robotic-based training translates into improved skill acquisition, accelerated learning curves, or superior surgical decision-making compared with traditional training models remains uncertain.

While exposure to robotic systems may contribute to a well-rounded educational experience, excessive reliance on these technologies could diminish trainee confidence with conventional instrumentation, raising concerns regarding training balance. Other evidence suggests that trainee participation in robotic-assisted TKA does not increase operative time, indicating that robotic platforms may support education without compromising efficiency; nevertheless, the broader educational impact of these systems warrants further investigation [15].

## Advancing three-dimensional balance in knee arthroplasty

Robotic assistance has demonstrated advantages in implant sizing, fit, and alignment [16]. Avoidance of component overhang reduces soft tissue irritation, while accurate sizing supports sagittal plane stability [9,16]. Sagittal alignment, historically one of the most challenging aspects of knee arthroplasty, can now be visualized and adjusted dynamically with robotic assistance [17]. In addition, robotic platforms enable real-time assessment of the interrelationship between femoral position, tibial slope, and component sizing and their collective influence on balance throughout the arc of motion [18].

Robotic assistance also highlights how adjustments made in one anatomical plane may produce subtle, interdependent changes in the remaining planes as a consequence of implant geometry. Although these effects may not be readily perceptible intraoperatively, they are mathematically inherent and may have implications for overall knee balance and joint kinematics.

Enhanced visualization of femoral rotational alignment further allows surgeons to anticipate downstream effects on medial–lateral balance and patellofemoral tracking, potentially reducing the need for lateral release and its associated morbidity [16,17]. Mid-flexion stability, which is closely linked to joint line restoration, may likewise be more reliably addressed with robotic assistance [17]. In the revision setting, robotic systems facilitate individualized 3-dimensional correction toward optimal component positioning across the sagittal, coronal, and axial planes, potentially reducing reliance on residual malposition or increased implant constraint to compensate for imbalance [9,17]. Nevertheless, the clinical significance of these technical advantages remains to be fully established, underscoring the need for long-term studies correlating improved balance metrics with patient-reported and implant-level outcomes.

## Extending benefits to total hip arthroplasty

Robotic technologies in total hip arthroplasty (THA) have increasingly garnered attention from implant manufacturers. Robotic assistance and related technologies have been promoted as potential solutions to persistent challenges in acetabular component positioning, as malposition remains common, with deviations from intended orientation reported in up to 50% of cases [19]. These challenges are further amplified in patients with obesity, complex anatomy, or abnormal spinopelvic mechanics. In posterior-approach THA, intraoperative pelvic motion of up to 15° has been reported, potentially compromising component accuracy and stability [20].

Robotic-assisted THA has been associated with improved acetabular component placement and may confer a protective effect against instability. In a single-center study, Bendich et al. reported a significantly lower risk of revision for dislocation following robotic-assisted THA, with an odds ratio of 0.3 compared with manual techniques [21]. Additional studies have demonstrated greater accuracy in achieving target acetabular alignment with robotic assistance, with significantly reduced variability in cup inclination and anteversion compared with conventional methods [22]. Furthermore, leg length discrepancy, among the leading causes of patient dissatisfaction and litigation following THA, remains a recognized limitation of conventional techniques [23].

Robotic assistance in hip arthroplasty enables real-time assessment of acetabular orientation, limb length, and femoral offset, while enhancing detection of impingement risk [24]. The ability to individualize component positioning relative to functional pelvic alignment may be particularly advantageous in patients with abnormal spinopelvic motion [24]. Nevertheless, continued investigation is required to determine whether these intraoperative advantages translate into lower dislocation rates, improve gait mechanics, and enhanced long-term implant survivorship.

## Precision in revision arthroplasty

Robotic assistance during revision knee arthroplasty represents an emerging application with the potential to address challenges such as bone loss, distorted anatomy, ligament attenuation, and retained hardware. Robotic systems allow integration of these variables to support implant positioning, augment preparation, and restoration of the joint line [17,25]. In contrast to conventional techniques, which often derive alignment from intramedullary stem position, robotic assistance may facilitate a more individualized reconstructive strategy by prioritizing balance and stability beginning at the restored joint line and progressing peripherally [17,25]. This “joint line-first” approach represents a potential departure from traditional revision philosophies and warrants further investigation to determine its clinical relevance.

Similarly, emerging applications of robotic assistance in revision THA may support restoration of the hip center of rotation and enhance preoperative planning of augment placement and screw trajectory, while also facilitating more consistent normalization of leg length and femoral offset to promote construct stability [26].

Early experience across revision applications suggests that robotic systems may improve intraoperative adaptability, particularly when unanticipated bone loss is encountered [17]. However, robust comparative studies are needed to determine whether robotic assistance in revision arthroplasty meaningfully reduces complications, improves functional outcomes, or better preserves bone stock compared with established revision techniques.

## Workflow, efficiency, and system-level considerations

Beyond precision, robotic platforms may also influence operative workflow and efficiency. Reductions in instrument tray utilization, standardized surgical workflows, and enhanced intraoperative decision support have the potential to decrease cognitive and physical demands on surgeons and operative teams [27]. These potential advantages are increasingly relevant as surgical volumes rise and concerns regarding surgeon workload and burnout persist. In addition, safety features such wrong-site alerts and virtual cutting boundaries may provide additional procedural safeguards.

Evidence regarding the effects of robotic assistance on operative efficiency and cost remains mixed. In a propensity-matched analysis, Tompkins et al. reported that robotic TKA performed by high-volume surgeons was associated with modestly longer operative times (approximately 8 minutes) and higher direct costs compared with conventional TKA, while length of stay and 90-day complication rates were similar and 30-day readmissions were lower [28]. In contrast, a large National Inpatient Sample study by Maman et al. demonstrated that robotic TKA was associated with shorter length of stay (1.9 vs 2.7 days) and lower rates of several in-hospital complications, albeit with higher overall hospital charges and a substantial increase in robotic utilization over time [29]. In hip arthroplasty, Zepeda et al. reported no significant difference in overall operative time between robotic-assisted and conventional posterior-approach THA; however, robotic assistance was associated with shorter acetabular reaming time [30].

Despite these potential advantages, logistical and economic challenges persist. Implant manufacturers may incur increased shipping and inventory costs to support robotic workflows; however, robotic platforms have demonstrated high accuracy in preoperative implant size prediction, often within one size in up to 98% of cases, which may help mitigate inventory burden [16]. Overall, the economic impact of robotic assistance, including effects on operative time, staffing requirements, supply chain logistics, and resource utilization, remains incompletely defined and warrants further investigation.

## Barriers and the imperative for evidence

The costs associated with the acquisition and maintenance of robotic systems remain among the primary barriers to widespread adoption [31]. Substantial capital investment, ongoing maintenance, disposable expenses, training requirements, and physical space constraints may limit feasibility, particularly for smaller institutions and ambulatory surgery centers. In addition, learning curves may initially increase operative times, and reimbursement policies remain inconsistent. Some robotic platforms require preoperative computed tomography (CT) imaging, raising concerns regarding radiation exposure and added cost; furthermore, if a CT scan is rejected or intraoperative CT registration fails, accuracy may be compromised, potentially leading to suboptimal outcomes. In contrast, imageless systems may offer lower-cost alternatives. Surgeons must also maintain proficiency with conventional techniques to ensure patient safety in the event of system malfunction or limited availability. Notably, only approximately half of insurance providers currently reimburse robotic-assisted procedures, often citing their designation as “experimental,” and some deny coverage for preoperative CT imaging, further constraining adoption [31].

Collectively, these barriers underscore the need for high-quality evidence to define value-based indications and guide responsible implementation of robotic technology. Although implant manufacturers increasingly promote robotics as a means of addressing intraoperative challenges, it remains unclear whether the associated upfront costs and time investment translate into clinically meaningful improvements in outcomes. Continued vigilance and well-designed comparative research are necessary to clarify the role of robotic assistance in arthroplasty and to ensure that adoption is guided by evidence-based decision-making focused on optimizing patient outcomes.

## Conclusion and future directions

Robotic assistance in hip and knee arthroplasty has progressed beyond novelty but has not yet reached full maturity. Emerging evidence suggests potential advantages in precision, reproducibility, and surgical education, with particular promise in complex primary and revision procedures [25,32]. However, important questions remain regarding long-term clinical outcomes, cost-effectiveness, and equitable access. Continued investigation through clinical trials, registry-based analyses, health-economic studies, and educational research is essential to refine robotic technology and more clearly define its role in contemporary arthroplasty. As demand for joint replacement continues to grow, rigorous, evidence-based evaluation will be critical to ensure that technological innovation translates into durable, high-quality outcomes across diverse practice settings.

## CRediT authorship contribution statement

**Matthew Bullock:** Writing – review & editing, Writing – original draft, Resources, Conceptualization. **Jonathan R. Danoff:** Writing – review & editing, Writing – original draft, Resources, Conceptualization.

## Conflict of interest

M. Bullock is on the speakers bureau/paid presentations for Smith & Nephew; is a paid consultant for Smith & Nephew; has stock or stock options in Stryker and Smith & Nephew; serves as an Associate Editor for *Journal of Arthroplasty andArthroplasty today*; is a board member/committee appointments for West Virginia Orthopaedic Society – AAHKS Digital Health and Social Media Committee; and has recused himself from the peer review process. J.R. Danoff is a paid consultant for Stryker, Surgical Specialties Corp, and Sanara Medtech; serves as an Associate Editor for *Arthroplasty today;* is a board member/committee appointments for AAHKS Committee; and has recused himself from the peer review process.

## Appendix A Supplementary data

Conflict of Interest Statement for Bullock

Conflict of Interest Statement for Danoff
