# Supplementary material for: Beyond Precision: Why the Future of Robotic Joint Arthroplasty Demands Continued Research
Source: Arthroplast Today. 2026 Jun 13;40:102071. doi: 10.1016/j.artd.2026.102071 (PMC13279439; doi:10.1016/j.artd.2026.102071)
Supplement: Conflict of Interest Statement for Danoff [file mmc2.pdf]

CONFLICT OF INTEREST STATEMENT

American Association of Hip and Knee Surgeons

(Adopted from the American Academy of Orthopaedic Surgeons disclosure statement)

The following form **must be filled out completely and submitted by each author (example, 6 authors, 6 forms). All items require a response. If there is no relevant disclosure for a given item, enter "*None.*"**

Manuscript Title: Improved Short-Term Outcomes in Robotic-Assisted Revision Total Knee Arthroplasty

1. Royalties from a company or supplier (The following conflicts were disclosed)

2. Speakers bureau/paid presentations for a company or supplier (The following conflicts were disclosed)

3A. Paid employee for a company or supplier (The following conflicts were disclosed)

3B. Paid consultant for a company or supplier (The following conflicts were disclosed)

Stryker, Surgical Specialties Corp, Sanara Medtech

3C. Unpaid consultants for a company or supplier (The following conflicts were disclosed)

4. Stock or stock options in a company or supplier (The following conflicts were disclosed)

5. Research support from a company or supplier as a Principal Investigator (The following conflicts were disclosed)

6. Other financial or material support from a company or supplier (The following conflicts were disclosed)

7. Royalties, financial or material support from publishers (The following conflicts were disclosed)

8. Medical/Orthopaedic publications editorial/governing board (The following conflicts were disclosed)

Arthroplasty Today

9. Board member/committee appointments for a society (The following conflicts were disclosed)

AAHKS Committee

Each author must sign AND print or type his/her name, date and submit a separate form

In addition, one BLINDED Conflict of Interest form (no author names used) should be submitted per manuscript with all author disclosures.

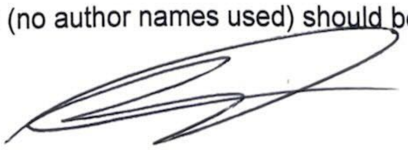

Jonathan Danoff, MD		7/11/25
Author Name (Print or Type)	Author Signature	Date
